# Intelligent Manufacturing of New Energy Vehicles and Financial Market Hedging Based on Soft Computing

**DOI:** 10.1155/2022/6593602

**Published:** 2022-04-04

**Authors:** Peizhi Zhao

**Affiliations:** Department of Accounting, Southampton Business School, University of Southampton, Southampton SO17 1BJ, UK

## Abstract

Globally, the energy supply in the market is tight and the oil price fluctuates sharply. With the increasing degree of environmental pollution, both developed and developing countries pay special attention to the development of new energy, and energy conservation and emission reduction have been put on the agenda. As new energy vehicles have the advantages of energy conservation and environmental protection, they are strongly supported by governments. Many countries regard new energy vehicles as substitutes for traditional vehicles, and their development has ushered in a good opportunity. Under the background that the government and enterprises pay more attention to the development of new energy vehicles, it is of positive significance to study the intelligent manufacturing enterprises of new energy vehicles in a province. This paper studies the manufacturing of new energy vehicles through soft computing. Next, this paper also analyzes the financial market. Enterprise risk is closely related to investor income and social healthy development, which is a hot issue concerned by the public and the government. However, in recent years, there have been a series of cases in which enterprises have suffered huge losses using “hedging” to avoid operational risks, which has aroused public concern about the motivation of enterprises to use derivative financial products. Based on the leverage characteristics of derivative financial instruments, scholars believe that the high leverage profit in the capital market is the main incentive for enterprises to use hedging for hedging speculation. Existing studies pay more attention to the consequences of enterprise hedging, and they analyze the impact of enterprise hedging, focus on the result level after enterprise hedging, and seek the significance of enterprise hedging. Through the research on soft computing, this paper will apply it to the intelligent manufacturing of new energy vehicles and promote the development of hedging in China's financial market

## 1. Introduction

After decades of development, the traditional automobile industry has become more technologically mature and has made great contributions to facilitating people's travel. However, with the development of the economy and the popularization of automobiles, the following two problems have also appeared: on the one hand, oil is the “blood” of the development of traditional automobiles, and it is a nonrenewable resource [1, 2]. If oil is used as the power source of automobiles for a long time, the development of cars becomes unsustainable, and the problem of fuel shortage is highly valued by governments of various countries [3]. On the other hand, traditional cars produce a lot of waste during use, causing serious damage to the environment, and car exhaust pollution has also been criticized by people [4, 5]. Environmentalists all over the world are calling for environmental protection and green and low-carbon travel [6]. Therefore, countries all over the world are paying more attention to energy conservation and emission reduction [7]. Combining the above two factors, to solve the problem of environmental pollution, new energy vehicles based on soft computing will be an important way to solve this problem, and it has also become an important direction for the green development and sustainable development of new energy vehicle intelligent manufacturing [8]. Next, this article analyzes the financial market. In the context of the integrated development of the global financial system, financial liberalization and economic globalization are becoming more important. China's financial market and the international financial instrument market are becoming closer [9]. Therefore, for Chinese companies, it is difficult to avoid risks caused by the price fluctuations of raw materials, products, and the financial environment [10, 11]. Companies are affected by the large fluctuations in the prices of bulk goods and upstream raw materials, and the company's profits have become more uncertain, causing huge losses to the company, and even endangering the company's operations [12]. Affected by the financial crisis and economic globalization, the price fluctuations in the bulk commodity market are not a short-term phenomenon [13]. How to deal with the impact of price fluctuations is the key to a company's sustainable operation [14]. For stable operations, companies should constantly adjust their business models to ensure that the importance of accurately identifying, grasping, and effectively avoiding risks brought by price fluctuations, and making effective use of the futures market for companies risks are hedged [15]. However, the huge losses caused by the use of hedging by enterprises are caused by the use of derivative financial instruments [16]. Based on the important management foundation of the enterprise, the perspectives of senior management and internal governance and the important external management foundation of the company are discussed from the macroperspective of the use of hedging [17]. Motivation issues help companies make decisions on derivative financial products, such as hedging, and avoid corporate risks [18].

## 2. Related Work

The literature shows the importance of studying the financial performance evaluation of new energy vehicles in a certain province and strives to provide suggestions for the development of the new energy vehicle industry in a province. Part of the research puts forward the improvement suggestions of the quality management team; the visual interface and system functions of the complete SPC software are designed. At the same time, it provides a software basis for the realization of the previously designed quality control system, also determines the thermal resistance of key parameters according to customer feedback and production performance, and determines the key process of heat sink dispensing process and DBC (ceramic copper sheet) cutting and mounting process. Through the 5M1E fishbone diagram analysis method, the cause of the defect was deeply analyzed, and the improvement plan, the adverse reaction plan, and the three-stage plan of SPC real-time monitoring were established. The SPC monitoring of thermal resistance parameters, glue quantity and DBC (ceramic copper sheet) placement pressure parameters, and other parameters were gradually realized, and the SPC control chart of thermal resistance parameters was drawn. There are also some literatures that propose the construction of a financial performance indicator system, which lays the foundation for the following AHP. The literature proposes to build a financial performance indicator system, which lays the foundation for the following level analysis. The literature proposes the construction of a financial performance indicator system, which lays the foundation for the analysis of the hierarchy below. At the same time, it pays attention to the benefits and problems of the company's hedging scheme, as well as the matters that should be paid attention to in hedging. And the Shanghai copper option is introduced, by designing two optimization schemes for hedging; one is using the Shanghai copper option alone and the other by combining the Shanghai copper future and the Shanghai copper option, and forecasting the scheme. After analyzing the advantages and existing problems of the G enterprise hedging scheme, it puts forward some matters that G enterprise should pay attention to in the future hedging. The literature proposes a method of matching car styling features according to user needs and builds a multilevel labeling car styling library. The literature shows case studies on user demand feature extraction, car styling feature matching, and car styling intelligent generation.

## 3. Soft Computing and Intelligent Manufacturing Technology of New Energy Vehicles

### 3.1. Soft Computing

Soft computing is a technique that increases actual uncertainty on the basis of hard computing to obtain an approximate solution or optimal solution to a practical problem to achieve the best solution. Soft computing technology can be applied to the research of product image design, and it can convert physical phenomena in a certain process or level of complex problems into mathematical models. At present, domestic and foreign scholars' research on soft computing methods mainly focuses on artificial intelligence and predictive models. In terms of product design, especially product images, there are few pieces of research on soft computing. The research on product image design based on computing methods in this article has certain theoretical and practical value.

**Figure 1:**
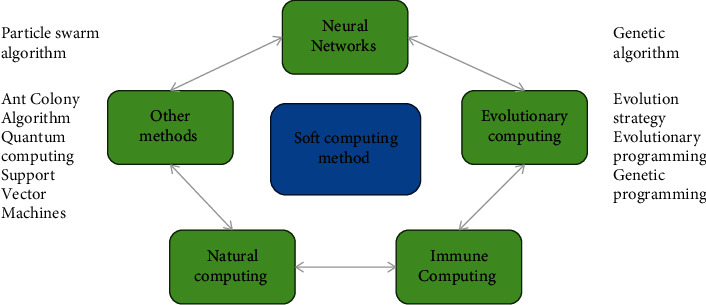
Soft computing method.

As shown in Figure 1, soft computing is a set of methods used to infer and learn human thoughts, process fitting models, and provide solutions to complex problems. It allows uncertainty and incomplete actual values to exist, and this method can better solve the uncertainty problem that is common in reality.

### 3.2. Intelligent Manufacturing of New Energy Vehicles

#### 3.2.1. Algorithm Model

The TFIDF algorithm is one of the classic algorithms of keyword extraction technology. The central idea is to extract keywords in the text according to the statistical information of word frequency and calculate the weight of the corresponding keywords. The high word frequency information of a certain keyword in the text and the low word frequency information of other texts reflect the keyword's ability to distinguish other texts. Therefore, keywords can be used as keywords in the text. The specific definition of TFIDF is in equations (1) and (2).

(1) $$\begin{array}{c} \left\{\begin{array}{c} {\text{TF}}_{ij}=\frac{n_{ij}}{\sum_{k}n_{kj}},\\ {\text{IDF}}_{i}=\log\frac{\left|D\right|}{\left|D_{i}\right|+1}, \end{array}\right. \end{array}$$

(2) $$\begin{array}{c} \left\{\begin{array}{c} w_{ij}={\text{TF}}_{ij}\times {\text{IDF}}_{ij},\\ w_{ij}=\frac{w_{ij}}{\sqrt{\sum_{k}w_{kj}}}. \end{array}\right.\; \end{array}$$

The principle of the cosine similarity calculation method is to use the cosine value of the angle between the two vectors in the vector space as the difference between individual objects. The specific definition is given in equations (3) and (4).

(3) $$\begin{array}{c} \left\{\begin{array}{c} A=\left\{a_{1},a_{2},a_{3},\ldots ,a_{n}\right\},\\ B=\left\{b_{1},b_{2},b_{3},\ldots ,b_{m}\right\}. \end{array}\right. \end{array}$$

From equation (3), we can see that *A* and *B* represent two independent objects, and each object contains *N*-dimensional features.

(4) $$\begin{array}{c} \text{sim}\left(A,B\right)=\cos\;\theta \frac{\overset{⟶}{a}\cdot \overset{⟶}{b}}{\left\|a\left|\cdot \right|b\right\|}. \end{array}$$

It can be seen from formula (4) that the method of calculating cosine similarity is to regard the object as a coordinate point in the *N*-dimensional coordinate system.

To continuously optimize the generated result image, a total loss function is defined, which is used to perform gradient descent to obtain a result image that matches the original image features. The formula of the total loss function is as follows:

(5) $$\begin{array}{c} L_{\text{total }}\left(\overset{⟶}{p},\overset{⟶}{a},\overset{⟶}{x}\right)=\alpha L_{\text{content }}\left(\overset{⟶}{p},\overset{⟶}{x}\right)+\beta L_{\text{style}}\left(\overset{⟶}{a},\overset{⟶}{x}\right). \end{array}$$

Among them, *L*_content_ is the content loss function, and *L*_style_ is the style loss function. The specific definitions are given in equations (6) and (7).

(6) $$\begin{array}{c} L_{\text{content }}\left(\overset{⟶}{p},\overset{⟶}{x},l\right)=\frac{1}{2}{\sum \left(F_{ij}^{l}-P_{ij}^{l}\right)}^{2}. \end{array}$$

Use the original image to calculate the convolution feature *P*_*ij*_^*l*^ and the convolution of the *l* layer.

(7) $$\begin{array}{c} L_{\text{style }}\left(\overset{⟶}{p},\overset{⟶}{x},l\right)=\frac{1}{4N_{l}^{2}M_{l}^{2}}{\sum \left(A_{ij}^{l}-G_{ij}^{l}\right)}^{2}. \end{array}$$

#### 3.2.2. Data Processing

Word frequency information is a measure of the frequency of words appearing in a document, and the specific definition is as follows:

(8) $$\begin{array}{c} p\left(s\right)=p\left(w_{1},w_{2},\ldots ,w_{n}\right)=\prod_{i=1}^{n}p\left(w_{i}|w_{1},w_{2},\ldots ,w_{i-1}\right). \end{array}$$

Mutual information statistics originally originated from the information theory, reflecting the connection between words. In the field of natural language processing, mutual information usually represents the internal cohesion between words, reflecting whether words can form new words. It is used to identify unregistered words and eliminate ambiguous words, and the specific definition of mutual information is as follows:

(9) $$\begin{array}{c} M\left(A,B\right)=\;\log_{2}\frac{P\left(A,B\right)}{P\left(A\right)P\left(B\right)}. \end{array}$$

However, upon considering the cohesion of the words alone, it is easy to split the words. The “keywords” in the “keywords” have a high mutual information value in the document, however, they are not unregistered words. Therefore, we need to introduce the concept of boundary freedom. The core idea of boundary freedom is to judge whether words can be used freely in each part of a sentence. Here, the minimum value of the number of words appearing on the left side of the phrase and the number of words appearing on the right side of the phrase is set as value of the boundary degrees of freedom. Equation (10) is defined as follows:

(10) $$\begin{array}{c} F\left(L,R\right)=F_{\text{minlen}}\left(L\left(w_{1},w_{2}\ldots w_{n}\right),R\left(w_{1},w_{2}\ldots w_{m}\right)\right). \end{array}$$

The word span reflects the description range of the descriptive vocabulary. The more the span, the more important the word and the stronger the whole. The calculation method is as follows:

(11) $$\begin{array}{c} W_{\text{span }}=\frac{\left|l_{i}\right|}{\left|L\right|}. \end{array}$$

Following the above-mentioned vocabulary and emotional feature factors, this article gives a definition of the TFIDF algorithm that integrates multiple features.

(12) $$\begin{array}{c} W_{ij}=\left(tf_{ij}\times idf_{ij}+W_{{\text{span}}_{j}}\right)\times W_{10c_{ij}}\times W_{{\text{speech}}_{ij}}\times M_{ij}. \end{array}$$

The effect of user demand extraction is composed of three evaluation indicators. The experiment uses fit, recall, and *F*1 value (*F*1-measures) to evaluate the effectiveness of keyword extraction. The calculation method is shown in formulas (13)–(15).

(13) $$\begin{array}{c} P=\frac{C_{\text{correct }}}{C_{\text{extract }}}, \end{array}$$

(14) $$\begin{array}{c} R=\frac{C_{\text{correct}}}{C_{\text{standard}}}, \end{array}$$

(15) $$\begin{array}{c} F_{1}-\text{Measure}=\frac{2pr}{p+r}. \end{array}$$

To verify the effectiveness of the fusion multifunctional TFIDF method proposed in this paper and the method of retrieving unregistered vocabulary, the PUBLIC-PRAISE dataset is used to test the experimental results of various vocabulary function combinations, and the specific experimental results are obtained using the experimental evaluation indicators, as shown in the Table 1 shown.

**Figure 2:**
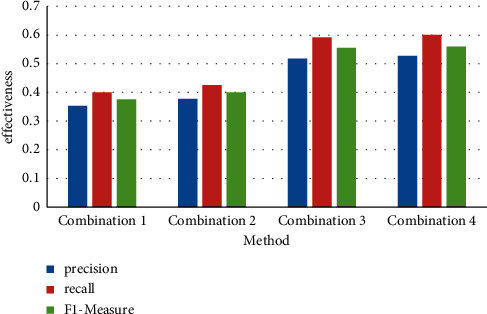
Comparison of TFIDF effects of different feature combinations.

The visualization result is shown in Figure 2.

**Figure 3:**
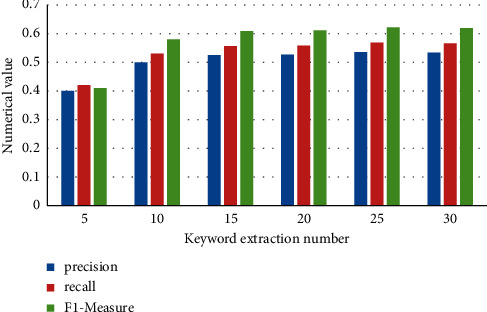
Comparison of the number of different keywords extracted.

To improve the performance of the keyword extraction effect of the method in this paper, the number of extraction of different keywords is set to *K* = 5, 10, 15, 20, 25, 30 for exploration, and the experimental results are shown in Figure 3.

The definition of bilinear upsampling is given in equations (16)–(18).

(16) $$\begin{array}{c} \left\{\begin{array}{c} f\left(R_{1}\right)=\frac{x_{2}-x}{x_{2}-x_{1}}f\left(Q_{11}\right)+\frac{x-x_{1}}{x_{2}-x_{1}}f\left(Q_{21}\right),\\ f\left(R_{2}\right)=\frac{x_{2}-x}{x_{2}-x_{1}}f\left(Q_{12}\right)+\frac{x-x_{1}}{x_{2}-x_{1}}f\left(Q_{22}\right), \end{array}\right. \end{array}$$

(17) $$\begin{array}{c} f\left(P\right)=\frac{y_{2}-y}{y_{2}-y_{1}}f\left(R_{1}\right)+\frac{y-y_{1}}{y_{2}-y_{1}}f\left(R_{2}\right), \end{array}$$

(18) $$\begin{array}{c} f\left(x,y\right)=\frac{f\left(Q_{11}\right)}{\left(x_{2}-x_{1}\right)\left(y_{2}-y_{1}\right)}\left(x_{2}-x\right)\left(y_{2}-y\right)+\frac{f\left(Q_{21}\right)}{\left(x_{2}-x_{1}\right)\left(y_{2}-y_{1}\right)}\left(x-x_{1}\right)\left(y_{2}-y\right) \end{array}$$

The optimized image *I*_*c*_^*∗*^ is initialized to *I*_*c*_^*∗*^ = *I*_*c*_ to facilitate subsequent optimization work and avoid local minima. To calculate the loss value of *I*_*c*_^*∗*^, *I*_*s*_, and *I*_*c*_, the definition of the loss function is shown as follows:

(19) $$\begin{array}{c} L_{\text{loss}}=\text{argmin}E_{s}\left(\phi \left(I_{c}^{*}\right),\phi \left(I_{s}\right)\right)+\alpha_{1}E_{c}\left(\phi \left(I_{c}^{*}\right),\phi \left(I_{c}\right)\right)+\alpha_{2}\gamma \left(I_{c}^{*}\right). \end{array}$$

*E*_*s*_ is the square of the Euclidean distance (*L*2 normal distance) between the optimized image layer and the corresponding feature layer of *I*_*c*_, i.e., the Markovian loss function defined as follows:

(20) $$\begin{array}{c} E_{s}\left(\phi \left(I_{c}^{*}\right),\phi \left(I_{s}\right)\right)=\sum_{i=1}^{m}\psi_{i}\left(\phi \left(I_{c}^{*}\right)\right)-\psi_{N\left(i\right)}{\left(\phi \left(I_{s}\right)\right)}^{2}. \end{array}$$

The calculation method of the optimal feature block is as follows:

(21) $$\begin{array}{c} N\left(i\right)=\text{argmin}\frac{\psi_{i}\left(\phi \left(I_{c}^{*}\right)\right)\cdot \psi_{j}\left(\phi \left(I_{s}\right)\right)}{\left|\psi_{i}\left(\phi \left(I_{c}^{*}\right)\right)\right|\cdot \left|\psi_{j}\left(\phi \left(I_{s}\right)\right)\right|}. \end{array}$$

To match the semantic label obtained by the semantic segmentation network with the result image of style transfer, the process uses the Hadamard image matrix to calculate the pixels between the semantic label and the result image. The calculation method is as follows:

(22) $$\begin{array}{c} R=Y\cdot Z. \end{array}$$

The background pixel retention rate is as follows:

(23) $$\begin{array}{c} I=\frac{N}{M}\times 100\%. \end{array}$$

The structure is similar.

(24) $$\begin{array}{c} \text{SSIM}\left(f,g\right)=L\left(f,g\right)gC\left(f,g\right)gS\left(f,g\right)\\ \left\{\begin{array}{c} L\left(f,g\right)=\frac{2\mu_{f}\mu_{g}+C_{1}}{\mu_{f}^{2}+\mu_{g}^{2}+C_{1}},\\ C\left(f,g\right)=\frac{2\sigma_{f}\sigma_{g}+C_{2}}{\sigma_{f}^{2}+\sigma_{g}^{2}+C_{2}},\\ C\left(f,g\right)=\frac{2\sigma_{f}\sigma_{g}+C_{2}}{\sigma_{f}^{2}+\sigma_{s}^{2}+C_{2}}. \end{array}\right. \end{array}$$

## 4. Financial Market Hedging Strategy and Optimization Research

### 4.1. Concept and Process

There are many types of hedging strategies, the most basic of which are long hedging and short hedging. The so-called long hedging is to buy derivative contracts at the moment when the spot price is expected to rise. The basis is that the derivatives and spot prices change in the same direction. When the spot price rises in the future, profits in the derivatives market are used to offset the spot market. Through this process, the purpose of hedging is achieved; the price forecast of short hedging is opposite to long hedging. At present, we mainly sell derivative contracts, however, the principle is the same as that of long hedging. They all use price fluctuations in the same direction. The principle is to achieve the purpose of using the derivatives market to avoid the price risk of the spot market.

In addition, there are initial assumptions in the initial selection and operation of the hedging strategy. If the hedger wants to hedge, he must conduct it in the spot risk exposure, and he can find a suitable time and amount of equivalent derivative products for his subject matter.

However, in the relationship between the actual spot market and the derivatives market, it is difficult to find derivatives that accurately correspond to the spot risk exposure, or there is a problem that there are no hedging instruments available in the derivatives market. In other words, the hedger needs to consider a cross-hedging strategy and should use another financial derivative that has a price correlation with the corresponding financial derivative.

The current real hedging in the derivatives market has also extended some more complex strategies based on long hedging and short hedging strategies. There are “rolling hedging” and “stripe hedging” in the short-term interest rate futures market. However, the core of these strategies is, in fact, still long and short strategies, which are only special forms extended under special circumstances. These expansion strategies have high requirements for use occasions. Hence, they can only solve the problems that exist in some special environments.

In the process of designing the actual hedging strategy, in addition to the principles of the derivatives market, it is also necessary to analyze the basic situation of the company itself. A more important aspect is the scope of hedging and the total amount of hedging. The scope of hedging refers to the company's outsourcing of raw materials, raw material production, raw material inventory, and spot products involved in product sales for hedging business operations through the corresponding futures varieties of the futures exchange.

**Figure 4:**
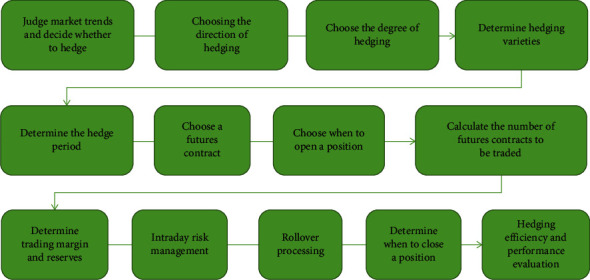
Hedging process.

The total amount of hedging includes the following parts: (1) the total amount of various specific types of hedging is limited to the company's total production of raw materials, total purchases of raw materials, and total product sales. (2) The total amount of various hedging operations shall not exceed the total amount limited by this plan; (3) When the hedging working group develops a hedging plan for specific project types, it will quantify step-by-step and implement step-by-step according to the actual situation and establish an optimized operating plan to allow the company's hedging. The period of preservation reaches the best state, as shown in Figure 4.

### 4.2. Strategy Classification and Evaluation

It is possible to conduct risk exposure analysis on the company's future raw material production, raw material procurement, product sales, and other business links to avoid the risks that market price fluctuations bring to the company's business.

The so-called unilaterally exposed enterprises are those with closed upstream and open downstream. On the contrary, the upstream and downstream enterprises are closed.

For companies with one-sided exposure, the focus of value preservation is the absolute price, and the price is unilaterally closed. Whether it is upstream or downstream, what the company has to do is to buy at the lowest possible price or sell at the highest possible price.

For this type of company, the basic method of hedging is relatively simple, either buying or selling, because one side of the price is fixed.

For companies with one-sided exposure, the uncertainty they face lies in the choice of projects, the choice of futures, the choice of the number of positions, and the choice of derivatives.

Therefore, the steps for a unilaterally exposed company to use the derivatives market for hedging can be mainly divided into three steps, which are as follows:

Firstly, determine the company's hedging target, then carry out price analysis and forecasting, and finally determine the hedging mode.

Different from unilateral exposure, bilateral exposure means that both the input price (cost) and output price change according to the price of the hedged object. This type of company usually comes from the middle reaches of the industrial chain, and the company's main business is trade or processing direction.

If the spot market price of the input and output products is the same, the company's core profit point is the difference between the processing fee and processing costs. In reality, the actual profit risk of the company lies in the price difference between the output product and the spot price of the input.

Since the actual spot target price usually fluctuates greatly, the value goal of the bilateral exposure company is to avoid the risk of the difference in the spot price of the sales item. In the actual hedging business, these companies are interested in the relative price of the spot rather than the absolute price of the spot target. The main purpose of the hedging plan is the hedging of the relative price.

According to the definition of hedging, an effective hedging operation must ensure that the combined benefits of the spot interest and the derivative market interest are positive, at least to ensure that the operation of the derivatives market allows the spot market to reduce losses.

In the current market, many companies do not know how to use hedging tools, especially after some state-owned enterprises have been involved in the “hedging” whirlpool of foreign futures markets. There are many doubts and negative comments in the market, which has led to the dismissal of companies that conduct hedging operations through the derivatives market.

In the actual operation of hedging companies, the evaluation of hedging effectiveness can be misleading. They may focus on the book profits and losses to evaluate the overall hedging effect. Obviously, this method is one-sided.

In addition, based on the correct hedging concept, the company can flexibly manage hedging based on actual risk exposure and cash flow in actual operations. It is not limited to copying and imitating the relatively successful models of other companies. Each company has its own special parts in terms of business conditions and financing methods, which need to be analyzed based on specific conditions, and different hedging models can be designed for different companies to better manage risk.

No matter how mature the company is, there is no guarantee that the actual hedging business will be perfect. There are uncertain factors, such as fundamental differences, cross hedging, and hedging costs. In addition to the company's natural profit-seeking nature, hedging operations are also the product of the game between controllable risk and excess profit, the product of the game between controllable risk and the maximum avoidance, and the reduction of basic differences and spread losses.

Therefore, when evaluating a company's hedging performance, firstly, determine the company's hedging target based on its own circumstances. In other words, to avoid spot market risks, the evaluation of the hedging effect should be based on whether to reduce risks and losses and how to operate the futures market. Loss can be reduced. Even if the risk exposure is not fully covered, hedging should be considered effective.

### 4.3. Effect Analysis

Table 2 shows the specific hedging strategies.

In early March 2020, according to the average price of nonferrous copper, the spot price of nonferrous copper was 45,390 yuan/ton. By May 2020, the spot price of nonferrous copper has fallen to 42,930 yuan/ton. Without hedging, a company's net asset loss would be RMB 12.3 million.

The specific calculation is as follows: a company's spot market profit and loss = (May-March copper market price) × company copper stock = (42930 yuan/ton − 45390 yuan/ton) × 5000 tons = 12.3 million yuan.

According to the aforementioned Shanghai copper futures hedging strategy, a company sold a total of 401 CU2005 and CU2006 contracts in early March. According to the set opening price, the average selling price in May was 44,601 yuan/ton, and the buying price was 42,861 yuan/ton and 42,721 yuan/ton.

According to calculations, the profit and loss of using the CU2005 contract is (44601 − 42861) × 2001 = 3.482 million yuan, the profit and loss when using the CU2006 contract is (44601 − 42721) × 2001 = 3.762 million yuan, the cumulative profit and loss is 7.244 million yuan, and the overall profit and loss situation is as shown in Table 3.

**Figure 5:**
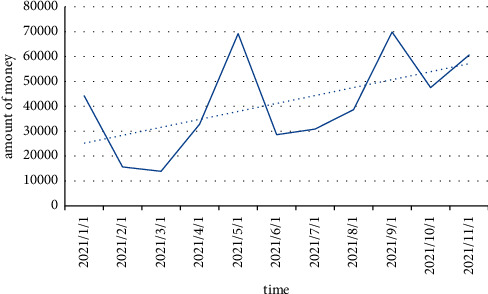
Corporate cash flow.

Taking the company's cash and cash equivalents as an indicator of the daily available cash flow, whether it is using Shanghai copper futures or Shanghai copper options for hedging, the funds occupied are covered by the company's daily operating cash flow, as shown in Figure 5. Cash flow pressure is relatively low.

According to the previous calculations, the hedging results, in this case, can be visually displayed, as shown in Table 4.

### 4.4. Optimized Plan Design

Shanghai copper options were listed for trading in September 2020. Like other commodity futures options, the basis of copper options contracts is copper futures contracts. Generally speaking, copper options are actually an option, i.e., traders can choose whether to withdraw their purchases at any time or the right to sell copper futures.

The general idea of using Shanghai copper options is similar to that of using Shanghai copper futures, both of which hedge the risk of falling copper prices while maintaining the potential for copper prices to rise. Therefore, the strategy of buying put options is adopted, the exercise price is set to 44,000–45,000, and the Shanghai copper options contract with the exercise price of 44,000 is finally selected, namely CU2005P44000.

Because of the low daily trading volume of Shanghai copper options, each order is limited to 100 lots, and the Shanghai copper options also use the bulk subscription method, with a total of 800 put options purchased.

When designing the hedging plan, the use of Shanghai copper options for hedging is another option for the company. It is mainly because Shanghai copper options use a lower option fee to obtain the future and buy and sell according to higher established prices. In the actual hedging process, because of the large trading volume and the difficulty of trading in the Shanghai copper options market, this option was not selected. However, in the following procedures, the benefits of this option can be analyzed through simulation, which can provide reference experience for the following design.

Because of the low daily trading volume of Shanghai copper options, the simulated backtesting process is difficult to meet the needs of one-time trading to cover the risk exposure. Hence, the transaction is carried out in batches, and the order is finally placed through 6 transactions. For the part of the transaction of 100 lots or more, the option premium is the average transaction price of the day.

According to the Shanghai copper option hedging strategy in the previous section, at the beginning of March, a company bought 800 lots of CU2005 P44000 at an exercise price of 44,000 yuan/ton. After the futures exercise, the partial profit and loss = (exercise price-CU2005 settlement on the exercise date) Price) × 4000 tons = (44000 yuan/ton − 41,725 yuan/ton) × 4000 tons = 9.1 million yuan, cumulative profit and loss = futures partial profit and loss-spot partial loss-option price = 9.1 million yuan − 12.31 million yuan − 428,800 yuan = −363.88 million yuan, the intuitive cost, and profit calculation are shown in Table 5.

The hedging optimization ideas in this paper have two main points: one is to use only Shanghai copper options and to introduce options that optimize the combination of Shanghai copper futures and options. The other is to consider whether the company can adopt the possibility of long-term hedging.

Although the optimization solution solved some of the problems in the case, it still exposed some risk points, which may require special attention in future operations.

First of all, in this case and optimization plan, the possibility of adjusting the production plan of the company was not considered. The company is at the downstream and end of the copper industry chain. The theoretical risk exposure should come from the upstream. Because of the adjustment of the production plan, the company's inventory is insufficient. To support daily production, the direction of hedging may be completely reversed. With the continuation of the current hedging direction, companies will also face losses.

In addition, because of the short hedging period of the company, neither the case nor the optimization plan has the expiry date of the derivative market contract, nor does it consider the risk of month-swapping and long-term coverage of the risk exposure. It is the hedging of other companies. Questions that cannot be skipped at the time. By comparing optimization plans and cases, we can understand the importance of derivatives trading volume in facilitating transactions, which also has a significant impact on the final hedging effect. If the trading volume in the options market is insufficient, the optimization plan has not been fully analyzed to support the possible consequences of hedging needs.

Neither the case nor the optimization plan considers other costs of the company's hedging, hedging expenses, other costs that need to be outsourced to specialized agencies, and other risks.

In addition, the company itself does not have a lot of hedging experience. Hence, it needs to seek the guidance of futures companies. There is a risk of leakage of the company's production and management secrets. Even if the company wants to carry out long-term hedging operations, it still needs training or employment and professional derivative personnel to design and manage its own hedging operations.

### 4.5. Analysis of Hedging Strategies in the Financial Market

In the process of using the derivatives market for risk management, companies establish a correct concept and make it clear that the original intention of the company to use derivatives is to avoid risks and not to profit through speculation. It requires a clear understanding of the risks that the company can bear. In the futures market, the high leverage provided by margin trading allows companies to support larger transactions with less capital. At the same time, it also introduces new risks and has higher requirements on whether companies understand the nature of futures market transactions.

Enterprise experience shows that the direction of the company's hedging may not match the company's position in the industry chain. The company is located in the downstream or terminal position of the copper industry chain. Traditional hedging thinking requires companies to hedge upstream risk exposures and is more sensitive to copper price increases. However, the company's inventory situation, in fact, is very good. For the company's daily production, it is more urgent to prevent inventory depreciation. In short, the company's actual risk exposure is similar to that of copper material manufacturers. What is worrying is that the price of copper has fallen. Therefore, the direction of hedging is opposite to the experience of hedging in the industry chain.

The derivatives market is the main place for companies to hedge, and it has an irreplaceable effect on companies to avoid market risks. However, it is undeniable that in the process of using the derivatives market, future market price fluctuations, margin risks, and liquidity risk may arise. Failure to correctly understand the existence of these risks will reduce the effectiveness of hedging and increase losses for companies.

As mentioned above, companies need to improve their professional knowledge and correctly understand the risks in the derivatives market. In addition to looking for specialized agencies to guide hedging, companies themselves also need to improve the professional quality of internal risk management institutions, fully understand the futures market, and improve the futures market. The risk identification and management functions enable the company's employees engaged in related businesses to strengthen their understanding of the futures market and strengthen the risk concept of all employees.

In addition, it should be recognized that under the current market environment and accumulated experience, risks in the derivatives market can be controlled by effective means, rather than allowing companies to withdraw.

Whether using futures or hedging options, capital occupation is inseparable. In the futures market, capital occupation is mainly reflected in the margin, while in the options market, capital occupation is mainly reflected in the option premium. Generally speaking, option costs are relatively fixed. Hence, the option use of a hedge fund can be determined at the time of trading. The futures market is traded on margin, and the amount of margin is much lower than the actual transaction amount supported. However, for companies with large positions, margin occupancy should also be considered.

## 5. Conclusion

The automobile industry is one of the important pillar industries of China's economy and has made great contributions to the vigorous development of China's economy. However, the future development of traditional automobiles has many limitations, and the new energy and green automobile industry are thriving with huge development potential, providing a strong driving force for promoting economic development. As new energy green vehicles are more energy-saving and environmentally friendly, the state and local governments provide fiscal and tax subsidies for the development of new energy vehicles, which brings many opportunities for the development of new energy vehicle intelligent manufacturing. This article starts with the company's actual hedging needs, analyzes the company's risk exposure, determines the hedging target, analyzes the copper spot market fundamentals, and predicts the price trend. In the process of simulating the company's hedging, we mainly evaluate the effect of hedging in terms of operability, current market profit and loss, and capital occupation. After evaluating and analyzing the case, this paper proposes an optimization plan based on the existing problems and conducts an evaluation of the backtest results.

## Figures and Tables

**Table 1:** Comparison of TFIDF effects of different feature combinations.

Method	Precision	Recall	Fl-measure
TFIDF	0.353	0.400	0.376
TFIDF + unregistered vocabulary	0.378	0.426	0.400
TFIDF + unregistered vocabulary + vocabulary features	0.518	0.592	0.555
TFIDF + unregistered vocabulary + vocabulary features + emotional features	0.528	0.601	0.560

**Table 2:** Hedging content of enterprises using Shanghai copper futures.

Item	Content		
Hedging purpose	Hedging the risk of 4,000 tons of standing inventory depreciation		
Contract selection	Shanghai copper 2005 and 2006 contracts		
Transaction direction	Sell		
Length of preservation	March–May		
Opening date	March	Closing date	May
Opening price range	44,000–45,000		
Number of contracts	800 hands		
Method of opening a position	Set up positions in batches, set up and sell 80 lots of Shanghai copper 2005 and 2006 contracts at 44,200, 44,400, 44,600, 44,800, and 45,000, respectively		
Program features	Avoid systemic risks to a certain extent, and retain the possibility of copper prices going up		

**Table 3:** The hedging effect of using Shanghai copper futures.

	CU2005	CU2006
Average selling price (yuan/ton)	44,601	44,601
Purchase price (yuan/ton)	42,861	42,721
Total number of contracts (hands)	401	401
Cover the risk part (tons)	2001	2001
Contract profit and loss (ten thousand yuan)	348.2	376.2
Total futures profit and loss (ten thousand yuan)	724.4	
Spot profit and loss (ten thousand yuan)	−1230	
Total profit and loss (ten thousand yuan)	−505.6	

**Table 4:** Comparison of the results of no hedging and hedging.

—	No hedging	Shanghai copper futures
Total profit and loss (ten thousand yuan)	−1230	−505.6
Capital occupation (ten thousand yuan)	0	C. 901

**Table 5:** Hedging backtest results using Shanghai copper options.

CU2005P44000 transaction situation	
Option fee (yuan/hand)	Volume (hands)
501	201
336	101
550	201
601	201
651	101

*Analysis of profit and loss results*	
Total option premium (ten thousand yuan)	42.88
Partial profit and loss of futures after exercise (ten thousand yuan)	910
Spot profit and loss (ten thousand yuan)	−1231
Accumulated profit and loss (ten thousand yuan)	−363.88

## Data Availability

The data used to support the findings of this study are available from the author upon request.
